# Supplementary material for: CAManim: Animating end-to-end network activation maps
Source: PLoS One. 2024 Jun 18;19(6):e0296985. doi: 10.1371/journal.pone.0296985 (PMC11185468; doi:10.1371/journal.pone.0296985)
Supplement: S1 File — (PDF) [file pone.0296985.s001.pdf]

# Supplementary Materials

## CAManim: Animating end-to-end network activation maps

Emily Kaczmarek[1,*], Olivier X. Miguel[2], Alexa C. Bowie[2], Robin Ducharme[2], Alysha L.J. Dingwall-Harvey[1,2], Steven Hawken[1,2,3,4], Christine M. Armour[1,5,6], Mark C. Walker[1,2,3,4,7,8,9,10], Kevin Dick[1,*]

**1** Children's Hospital of Eastern Ontario Research Institute, Ottawa, Canada
**2** Clinical Epidemiology Program, Ottawa Hospital Research Institute, Ottawa, Canada
**3** School of Epidemiology and Public Health, University of Ottawa, Ottawa, Canada
**4** ICES, Toronto, Canada
**5** Department of Pediatrics, University of Ottawa, Ottawa, Canada
**6** Prenatal Screening Ontario, Better Outcomes Registry & Network, Ottawa, Canada
**7** Department of Obstetrics and Gynecology, University of Ottawa, Ottawa, Canada
**8** International and Global Health Office, University of Ottawa, Ottawa, Canada
**9** BORN Ontario, Children's Hospital of Eastern Ontario, Ottawa, Canada
**10** Department of Obstetrics, Gynecology & Newborn Care, The Ottawa Hospital, Ottawa, Canada

¤Current Address: Children's Hospital of Eastern Ontario Research Institute, Ottawa, Ontario, Canada * {ekaczmarek, kdick}@cheo.on.ca

**This document contains the supplementary materials in support of the main manuscript. Links to supporting code and datasets are made publicly available for use by the research community.**

# Code and Data Availability

Publicly available examples of CAManim are available at:
https://omni-ml.github.io/pytorch-grad-cam-anim/intro.html

The codebase implementing the CAManim method for animating end-to-end network activation maps is available at: https://colab.research.google.com/github/OMNI-ML/pytorch-grad-cam-anim/blob/adapt-basecam-to-support-cam_anim/tutorials/_CAManim_animating_end2end_activation_maps.ipynb

A Google Collab notebook demonstrating the use ov CAManim on the Mednist dataset using a DenseNet network is available at:
https://colab.research.google.com/github/OMNI-ML/pytorch-grad-cam-anim/blob/adapt-basecam-to-support-cam_anim/tutorials/CAManim_mednist_tutorial.ipynb
